# Evaluation of the Effect of *Limosilactobacillus fermentum* CECT5716 on Gastrointestinal Infections in Infants: A Systematic Review and Meta-Analysis

**DOI:** 10.3390/microorganisms9071412

**Published:** 2021-06-30

**Authors:** Belén Pastor-Villaescusa, Ruth Blanco-Rojo, Mónica Olivares

**Affiliations:** 1 Metabolism in Childhood Research Group, Maimonides Biomedical Research Institute of Cordoba (IMIBIC), Reina Sofia University Hospital, University of Cordoba, 14004 Córdoba, Spain; belen.pastor@imibic.org; 2 Biosearch Life SA, Camino de Purchil 66, 18004 Granada, Spain; rblanco@biosearchlife.com

**Keywords:** gastrointestinal infection, diarrhea, formula-fed infant, probiotics, *Lactobacillus fermentum*, *Limosilactobacillus fermentum*

## Abstract

Reducing the incidence of gastrointestinal infections (GIs) that occur at early stages to mitigate hospitalizations and treatments with adverse effects is a promising strategy for providing well-being to infants and their families. This systematic review and meta-analysis explores whether the early administration of *Limosilactobacillus fermentum* CECT5716 might be effective as a preventive therapy for GIs. We reviewed the literature to identify randomized controlled trials (RCTs) investigating the effectiveness of milk formulas supplemented with *L. fermentum* CECT5716 administered to infants at early stages to reduce the incidence of GIs. The MEDLINE (via PubMed), Web of Science (WoS), and Cochrane Central Register of Controlled Trials (via CENTRAL) databases were searched up to 15 June 2021. GI data from the included studies were synthesized in a random-effects model. Three RCTs were finally selected including 435 infants. There was a significant reduction in the incidence rate of GIs for those receiving *L. fermentum* CECT5716 compared with those receiving placebo (IRR: 0.52, 95% CI: 0.36–0.74, *p* = 0.0004). Heterogeneity between studies was moderate (I^2^ = 54.5%). Based on the present systematic review and meta-analysis, the administration of *L. fermentum* CECT5716 at doses from 1 × 10^9^ to 8.4 × 10^8^ cfu/day in milk formulas may prevent GIs in infants up to 12 months old. Longer-term studies including a higher number of infants are needed to determine whether the use of this probiotic during the early stages of life is an efficient way to reduce the incidence of GIs.

## 1. Introduction

Although breast milk was once considered a sterile fluid, it is now widely accepted that it has its own unique microbiome, consisting of many commensal bacteria [1,2]. Most studies report that the stool microbiota of breastfed infants differs from that of infants fed formula [3]. The lactic acid bacteria present in human milk, in addition to other bifidogenic compounds such as oligosaccharides, are transferred from the mother to the infant through lactation [2], which may, at least in part, be responsible for some of the beneficial effects observed in breastfed infants [4]. Hence, there is a large body of evidence documenting the benefits of human breast milk in infants, including reduction of morbidity and mortality and protection against specific infections during the breastfeeding period [5,6]. This fact is extremely relevant for public health, because infectious diseases are the most common type of illness among infants worldwide. Thus, international guidelines recommend exclusive breastfeeding for all infants in the first six months of life, as it provides the best nutritional start for infants and promotes their healthy growth and development [7].

Unfortunately, exclusive breastfeeding is not always possible. Accordingly, formula milks are increasingly being supplemented with probiotics, prebiotics, or symbiotics to achieve a similar intestinal microbiota composition in formula-fed infants to in breast-fed children [4]. The European Society of Pediatric Gastroenterology, Hepatology, and Nutrition (ESPGHAN) Committee on Nutrition states that supplementation of infant or follow-on formulas with a few probiotics may be associated with a reduction in the risk of nonspecific gastrointestinal infections (GIs) as well as antibiotic use [8].

Scientific evidence supporting the use of probiotics for the prevention of infectious diseases is only emerging, though the results appear to be promising. Previously known as *Lactobacillus fermentum* CECT5716, it is currently named, according to the recent nomenclature, *Limosilactobacillus fermentum* CECT5716 [9]. This probiotic is naturally present in breast milk [10] and it has been proposed as a probiotic for formula milk due to its safety and functional [11], anti-infectious [12], immunomodulatory [13], and anti-inflammatory [14,15] properties. This strain is also able to colonize the mammary gland when administered orally in a capsule to lactating mothers [16]. Additionally, such supplementation is considered an efficient strategy for the treatment and prevention of infectious mastitis during lactation [16,17,18].

In this study, we thoroughly examined the effect of *L. fermentum* (CECT5716 Lc40) on the incidence rate of GIs in infants using a systematic review. Subsequently, we aimed to statistically guarantee the robustness of the evidence through synthesized the data from the clinical studies in a meta-analysis.

## 2. Materials and Methods

This review was conducted following the Preferred Reporting Items for Systematic Reviews and Meta-Analyses (PRISMA) statement (Supplementary Material S1) [19].

### 2.1. Eligibility Criteria

This work considered randomized controlled trials (RCTs) evaluating the effectiveness of *L. fermentum* CECT5716 for the prevention of GIs in infants in comparison with placebo. To be selected for analysis, studies had to meet the participants, intervention, control, outcome, and study design (PICOS) criteria [20]. Hence, eligible populations were healthy female and male infants who were being fed infant formula. Ethnicity was not an inclusion criterion. All doses and forms of *L. fermentum* CECT5716 administration were included. The GI incidence was the main outcome. Regarding the study design, prospective, parallel, and crossover RCTs were included. The study sample size was not restricted. To be considered for the full-text screening phase, the abstract had to be available in English or Spanish. Conference abstracts, case reports, ecological studies, and letters to the editor were excluded, as were those studies with cohort or case-control design and those analyzing the effect of other probiotics and/or bioactive compounds as the only intervention.

### 2.2. Search Strategy

We conducted a systematic, computerized literature search from inception until 15 June 2021 in the following three electronic databases: MEDLINE (via PubMed), Web of Science (WoS), and Cochrane Central Register of Controlled Trials (via CENTRAL). We created a search strategy combining index terms and keywords related to (1) “Lactobacillus fermentum CECT5716, Limosilactobacillus fermentum”, (2) “gastrointestinal infections”, and (3) “childhood”. No filters were applied to ensure the sensitivity of the search. The detailed search strategies used for the respective databases are presented in Supplementary Material S2. The reference lists of the included studies and similar reviews were hand-searched independently by two reviewers (B.P.-V. and R.B.-R.) to identify additional studies. Finally, complementary internet searches were conducted, and two trial registries (*ClinicalTrials.gov*: http://www.clinicaltrials.gov, accessed on 15 June 2021, and EU Clinical Trials Register: http://www.clinicaltrialsregister.eu, accessed on 15 June 2021) were additionally revised.

### 2.3. Study Selection

After the removal of duplicate studies, titles, abstracts, and full texts were screened according to the aforementioned selection criteria. Two researchers (B.P.-V. and R.B.-R.) completed all steps of the screening process independently, and discrepancies were resolved by discussion; if necessary, another reviewer was consulted (M.O.). During the full-text screening, a list with references not meeting eligibility criteria was kept, with notes for the reasons for exclusion. Data on the study design, population characteristics, exposures, comparator/control groups, outcomes, statistical methods, and results were assessed independently by two reviewers (B.P.-V. and R.B.-R.). Differences were resolved by discussion.

### 2.4. Data Extraction and Assessment of the Risk of Bias

Cochrane Review Manager (RevMan) (Computer program, Version 5.4. Copenhagen: The Nordic Cochrane Centre, The Cochrane Collaboration, 2011) was used for data extraction. Data for the authors, year of publication, language, study characteristics, participant description, intervention characteristics, outcomes, and statistical analysis were extracted independently by two reviewers (B.P.-V. and R.B.-R.). Differences were resolved by discussion. The number of GI events was used as the unit of analysis. For studies with more than one intervention arm, only arms relevant to the review and meeting the inclusion criteria were extracted. For missing data, authors of eligible RCTs were contacted via email. The most relevant data are summarized in Table 1. The reviewers independently, but without being blinded to the authors or journal, assessed the risk of bias in the studies that met the inclusion criteria. A summary graphic of the risk of bias assessment was created using RevMan, Version 5.4 (The Nordic Cochrane Centre, Copenhagen, Denmark), which includes the following criteria: adequacy of sequence generation, allocation concealment and blinding of participants, personnel and outcome assessment, extent of loss to follow-up, i.e., the proportion of patients for whom the investigators were not able to determine outcomes (incomplete outcome data), and other biases not classified (e.g., use of invalidated outcome measures) that must be reported. The classification was low risk of bias or high risk of bias and unclear bias when the study did not provide sufficient data to assess the bias risk [21].

### 2.5. Data Synthesis

We pooled data from the trials we judged to be clinically homogeneous using RevMan 5.4, and meta-analyses were performed with STATA v.14.0 software (Stata Corp LLC, Lakeway Drive, College Station, TX, USA). Dichotomous outcomes from the results of individual studies and pooled statistics are reported as the incidence rate ratio (IRR) between experimental and control groups with the 95% confidence interval (95% CI). The IRR and 95% CI were extracted from individual studies and combined to obtain a pooled IRR and 95% CI by the generic inverse variance method. As heterogeneity among the studies was suspected, a random-effects model was applied. Heterogeneity was quantified by I^2^ (interpreted as the percentage of the total variation between studies attributable to heterogeneity rather than to chance). We considered an I^2^ statistic value of 0% to 40% as low heterogeneity, 41% to 60% as moderate heterogeneity, 61% to 90% as substantial heterogeneity, and over 91% as considerable heterogeneity [20].

### 2.6. Quality of the Evidence

We graded the strength of the body of evidence for the effect of *L. fermentum* CECT5716 on the incidence rate of GIs in infants using the criteria of the Grading of Recommendations Assessment, Development, and Evaluation (GRADE) method [22], as modified by the Cochrane Handbook for Systematic Reviews of Interventions [23]. This approach assesses five key domains: risk of bias, heterogeneity, directness, precision of the evidence, and risk of publication bias. We separately report the applicability of the body of evidence based on descriptions of the populations, interventions, comparators, duration of study, and settings of the included studies. To evaluate publication bias, we intended to use a test for funnel plot asymmetry offered by [24]; however, publication bias was not formally assessed using a funnel plot due to the small number of studies (<10) included in the meta-analysis.

## 3. Results

**Figure 1:**
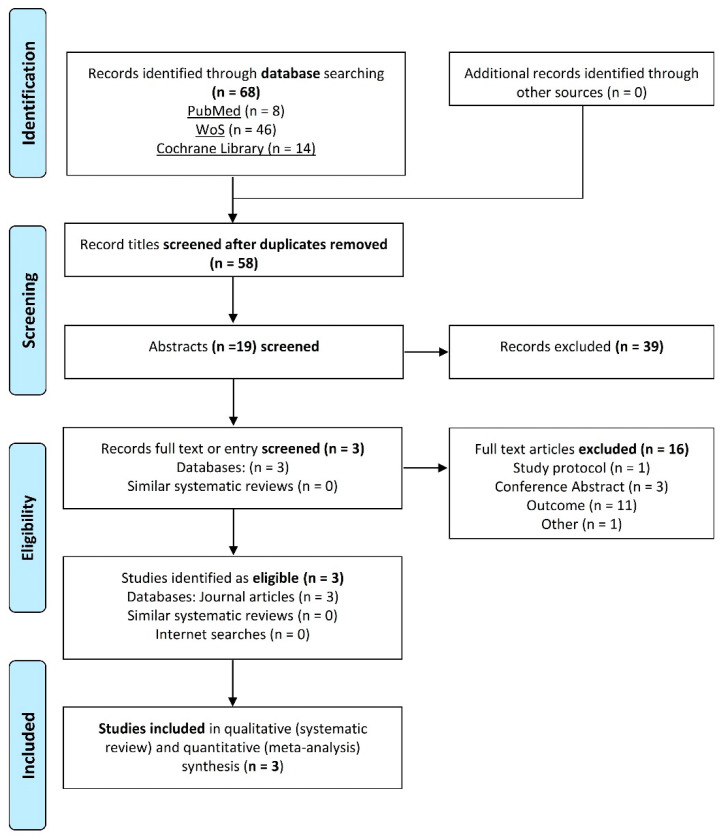
PRISMA flow diagram illustrating the selection process of included studies.

Figure 1 shows the RCTs retrieved and selected through the literature search. A total of 68 records were obtained using the equation proposed in the different databases (MEDLINE, WoS and Cochrane). Following the removal of duplicates, 58 references were included in the title screening. The topic under review was not addressed by 39 abstracts, and these studies were therefore excluded. The abstracts of the remaining 19 publications were screened for eligibility following the PICOS criteria. Articles that did not follow the PICOS criteria, study protocols, or conference abstracts were excluded. Ultimately, three publications were considered suitable for a detailed evaluation of full-text reports and included in the systematic review and meta-analysis [4,25,26].

### 3.1. Study Characteristics

The characteristics of the included RCTs are presented in Table 1. The included randomized trials of a total of 512 infants, of whom 435 were followed up. All included studies were double-blind, randomized, controlled trials and published in English. All studies were conducted in Spain [4,25,26]. Two were published in 2012 [4,24], and the other was published in 2019 [25]. All the studies included healthy infants exclusively fed formula, with an age ranging from one month [4,25] to six months [24]. The diagnosis of infectious disease was made by the pediatrician based on specific symptoms and standardized definition. The primary outcome evaluated in Gil-Campos et al. and in Maldonado et al. (2019) was the average weight gain between baseline and four months of age [4,25]. In both studies, the incidence of GIs was one of the secondary outcomes, as defined as loose or watery stools ≥3 times/day, with or without fever or vomiting [4,25]. However, in Maldonado et al. (2012) (infants age: 6–12 months), the incidence of GI (as a primary outcome) was the occurrence of loose or watery stool ≥4 times/day, with or without a fever or vomiting [24].

The daily dose of *L. fermentum* CECT5716 administrated was 8.4 × 10^8^ colony-forming units (cfu) in Gil-Campos et al. [4], 1 × 10^9^ cfu up to six months, and 7–8 × 10^8^ cfu between six and 12 months in Maldonado et al. (2019) [25] and 2 × 10^8^ cfu in Maldonado et al. (2012) [24]. The duration of the intervention period ranged from five to 11 months. In all the studies, *L. fermentum* CECT5716 was administered as an ingredient of a standard powdered infant formula with a nutritional composition in accordance with current EU regulations. Maldonado et al. (2012) [24] and Gil-Campos et al. [4] supplemented infant formula with galactooligosaccharides (GOSs) (0.4 and 0.3 g/100 mL, respectively) along with probiotics to provide a symbiotic mixture; their control formula also contained GOSs in the same amount. Follow-up visits were performed by pediatricians at baseline and every two months in the case of Gil-Campos et al. and at baseline and every three months in Maldonado et al. (2012) and (2019) [24,25].

The concentration of the probiotic in the formula was analyzed and confirmed every two months in the three RCTs. Regarding safety, all formulas were well tolerated, and compliance was good. Furthermore, no adverse effects related to formula consumption were reported.

### 3.2. Overall Results

**Figure 2:**
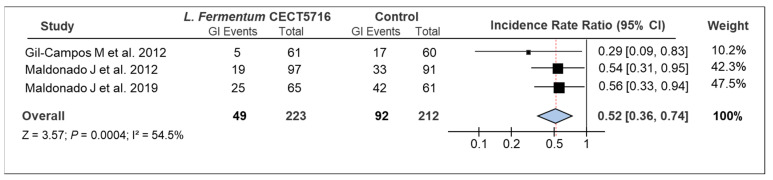
Forest plots illustrating the effect of *L. fermentum* CECT5716 interventions on the incidence of GIs in formula-fed infants. Abbreviations: CI, confidence interval; GI, gastrointestinal infection.

GI data from the three included studies were synthesized in a random-effects model. A forest plot shows the results of the analysis, with the sample size and number of GI events for each group in each study (Figure 2). Additionally, the IRR with 95% CI were noted, with the incidence rate of GIs being lower in the intervention group in all studies. Pooled results showed a significant reduction in the incidence rate of GIs in children receiving *L. fermentum* CECT5716 with children receiving the placebo (n = 435, IRR: 0.52, 95% CI: 0.36–0.74, *p* = 0.0004). Heterogeneity between studies was moderate (I^2^ = 54.5%) (Figure 2).

### 3.3. Risk of Bias Assessment

**Figure 3:**
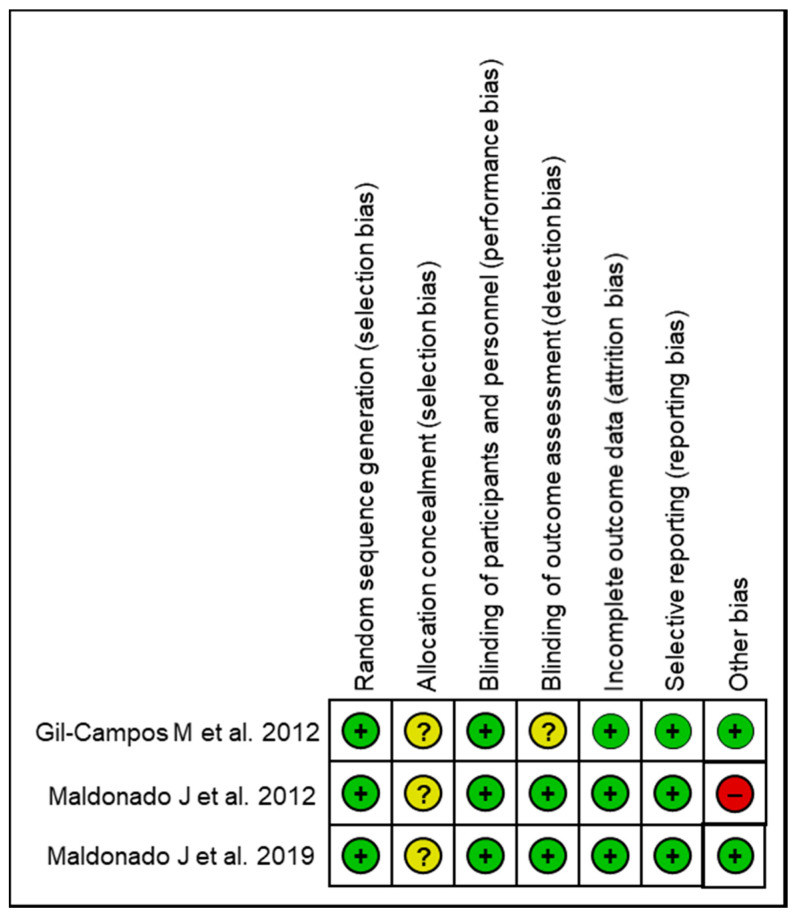
Summary of risk of bias assessment of included studies. Green (+): Low risk; Red (−): High risk; Yellow (?): Unclear risk.

The results of the bias risk assessment of the included studies are summarized in Figure 3. None of the included studies were rated as having a low risk of bias for all items of the assessment tool. Two studies received no high risk of bias ratings [4,25]. All studies were found to have employed adequate random sequence-generation methods and suitable methodology for the blinding of participants and personnel, clearly described all missing data (if any), and reported all outcomes (Figure 3). Only one study presented incomplete information about the blinding of the outcome assessment [4]. In all three studies, insufficient information on allocation concealment was provided. Finally, one study presented a high risk of bias because of a lack of declared conflict of interest of one author who was an employee of the company that funded the RCT [24].

### 3.4. GRADE Assessment

All the studies presented in this meta-analysis were RCTs, which are considered to provide the highest quality of evidence; however, the quality of the body of evidence was considered moderate. As indicated in the risk of bias assessment, only one study presented one of the evaluated items as having a high risk of bias; thus, we considered that there were no limitations that may bias estimates of the treatment effect. The number of studies included in the meta-analysis hindered the performance of subgroup analyses to identify a plausible explanation for the moderate heterogeneity found, prompting the downgrading of the strength of the body of evidence from high to moderate. Moreover, the evidence was direct because all the studies presented data on GI events diagnosed by a pediatrician based on specific symptoms and standardized definitions. The precision of the effect estimate was assessed by considering the 95% CI, which was found to be of moderate width. Although publication bias was not formally assessed using a funnel plot due to the small number of studies, there was a low probability of unreported studies due to the specificity of the review question.

## 4. Discussion

The current systematic review examined RCTs evaluating the effect of *L. fermentum* CECT5716 on the incidence rate of GIs in infants. Meta-analysis showed evidence for a significant preventive effect of *L. fermentum* CECT5716 administration against GIs. A significant reduction of 48% in the incidence rate in infants receiving *L. fermentum* CECT5716 was reported in comparison with those receiving the placebo (IRR: 0.52, 95% CI: 0.36–0.74, *p* = 0.0004). Gil-Campos et al. reported the largest significant finding, with a reduced incidence of diarrhea of 71% compared with the control group (IRR: 0.29, 95% CI: 0.09–0.83, *p* = 0.018) [4]. Furthermore, Maldonado et al. (2012) showed a significant reduction of 46% compared with controls (IRR: 0.54, 95% CI: 0.31–0.95, *p* = 0.032) [24]. Finally, Maldonado et al. (2019) reported similar data, with a reduction of 44% vs. controls (IRR: 0.56, 95% CI: 0.33–0.94, *p* = 0.014) [25]. Moreover, the lack of a high risk of bias identified during the assessment increased confidence in the findings of these RCTs.

GI is the major cause of acute diarrhea and has been proposed as an important target for the beneficial effects of probiotics on pediatric diseases [27,28]. Diarrhea, as one of the gastrointestinal disorders that commonly occurs, causes distress to infants and parents and leads to a cascade of discomfort, with repetitive consultations with health care providers [29]. Diarrhea is a major infectious cause of childhood morbidity and mortality worldwide [30] with an emotional and economic burden for the families of affected children, as well as a burden on society [31]. In fact, reports of GIs in many industrialized countries continue to increase and are largely related to waterborne and foodborne outbreaks [32], which can cause pediatric caregivers to misuse primary care and GI specialized care, driving unnecessary investigations and pharmacological treatments [33].

Over the last few years, there have been an increasing appreciation and emphasis on promoting breast milk feeding to enhance infant health, growth, and development. Indeed, as a source of commensal bacteria that further enhanced infant health, breast milk offers further benefits associated with breastfeeding, including protection against pathogens, promotion of gut colonization by beneficial microbes, and, therefore, reduced incidences of gastrointestinal disease [34]. The isolation of potential probiotic strains from milk has been the focus of some investigations [12,35]. Overall, the early stages of life represent an opportunistic period in which the gut microbiota may be more prone to changes caused by interventions involving probiotics, prebiotics, or their combinations [36]. Their administration during the perinatal period and lactation to favor infant gut colonization with potentially beneficial bacteria has been proposed based on the strength of evidence that bacteria are transmitted from mother to neonate through direct contact with the maternal microbiota during birth and through the supply of breast-milk bacteria during lactation [37,38]. Nevertheless, although probiotics may have preventive or therapeutic effects against diarrhea of various etiologies, not all probiotics are effective, and physicians must select preparations with proven efficacy [39]. *L. fermentum* CECT5716 is a probiotic strain originally isolated from four-day postpartum human milk [40] and is therefore naturally found in human breast milk. Different mechanisms, including competitive phenomena, production of antibacterial compounds, and improvement of the immune response, have been attributed to the anti-infectious and bactericidal activities of *L. fermentum* CECT5716 [12,13].

Furthermore, the beneficial properties of prebiotics such as oligosaccharides mainly relay on their ability to modulate the gut microbiota composition and to generate fermentation products (short-chain fatty acids), providing anti-inflammatory and anti-infectious properties [41]. The administration of GOS along with the probiotic, both in Gil-Campos et al. [4] and Maldonado et al. (2012) [25], may explain a synergistic effect enhancing the beneficial effect of *L. fermentum* CECT5716. However, it is important to highlight that, in these studies, the control formula also presented GOS, and therefore the potential effect that was observed is, at least mainly, exerted by the probiotic.

Regarding other probiotic strains, controversial findings have been reported. In some studies, *Bifidobacterium animalis* subsp. *lactis* BB-12 was administered (10 billion cfu/day) to 1-month-old infants for eight months [42] and for two years [43] to evaluate impacts on the risk of acute infectious diseases. The authors reported no significant differences compared with controls regarding the incidence of gastrointestinal infections, in accordance with the findings reported in other RCTs combining *B. animalis* subsp. *lactis* BB-12 with prebiotics [44]. In an RCT involving children aged six to 59 months with severe acute malnutrition, the combination of *B. animalis* subsp. *lactis* and *Lactobacillus rhamnosus* (10 billion cfu, 50:50) for 8–12 weeks had no effect on reducing diarrhea episodes during hospitalization, though it did reduce the number of days with diarrhea by 26% in outpatient treatment [45]. Other studies administrating the same combination of probiotics did not report a significant reduction in the incidence of GIs during the first seven months of life in 2-month-old infants [46] or after six months in infants aged 8 to 14 months [47]. For *Lactobacillus plantarum* DSM9843, no beneficial effect was observed for the incidence of gastrointestinal symptoms in children who were prescribed antibiotics [48]; nor in *B. lactis* CNCM I-3446 in C-section delivered infants [49] or combined with oligosaccharides in full-term infants [50]. Nevertheless, another RCT involving healthy term infants 4 to 10 months old found that those fed a formula supplemented with *L. reuteri* or *B. lactis* BB-12 had fewer and shorter episodes of diarrhea compared with controls after 12 weeks [51]. Furthermore, a meta-analysis of three RCTs performed to assess the efficacy of *L. rhamnosus* administration to prevent healthcare-associated diarrhea concluded that compared with the placebo, this probiotic has the potential to reduce the overall incidence of healthcare-associated diarrhea, including rotavirus gastroenteritis [52]. According to the report published by the ESPGHAN Committee in 2016, the use of *L. rhamnosus* is recommended for preventing antibiotic-associated diarrhea in children [53]. Based on the data presented herein, *L. fermentum* CECT5716 is another promising candidate to reduce the incidence of GIs in 1- to 12-month-old infants.

## 5. Strengths and Limitations

Our systematic review has several strengths. The review was based on the methodology developed by Cochrane Collaboration and reported according to the PRISMA statement, and we sought to decrease the risk of bias (i.e., no filters were applied during the search strategy, attempts to identify unpublished trials). The risk of bias in the included trials was also assessed. Finally, our review focused on a single probiotic strain; thus, the findings are clinically relevant and applicable to practice. Nevertheless, the current meta-analysis has some limitations. First, only a small number of studies were available. However, all included studies were RCTs with more than 100 participants, which increased the methodological quality of the body of evidence, as outlined in the GRADE assessment [22]. Second, the unexplained moderate heterogeneity found downgraded the quality of the evidence. Another limitation is the fact that one of the studies [24] used a different dose of the probiotic compared to the other two [4,25]. Although some studies found a relationship between dose and the observed effect for other probiotic strains [54,55], the results observed in this meta-analysis did not support a dose–response effect for *L. fermentum* CECT5716. This observation is also in agreement with the nondependent dose effect exerted by *L. fermentum* for other applications [16]. Finally, although GI was diagnosed by a pediatrician in all three studies, the definition of diarrhea was not homogeneous. Indeed, the Consensus Group on Outcome Measures Made in Pediatric Enteral Nutrition Clinical Trials (COMMENT) agreed that consensus for a core set of outcomes with agreed definitions, including those related to acute diarrhea, should be reached and that these outcomes should be measured and reported in nutritional trials [56].

## 6. Implications

In Europe, it is estimated that the incidence of acute GI ranges from 0.5 to 2 episodes per child per year in those under the age of three [57]. Effective preventive strategies for this pathology, which is a major reason for hospitalization in this age range, are required to improve infant health. The data reported are promising, and early administration of *L. fermentum* CECT5716 might be effective as a preventive therapy for GI.

Furthermore, breastfed infants have a lower incidence of infections than those fed with formula. Hence, formula milk is increasingly being supplemented with probiotics, prebiotics, or symbiotics to achieve an intestinal microbiota in formula-fed infants similar to that in breastfed infants. The current meta-analysis strengthens the evidence with regard to the effect of formula milk supplemented with *L. fermentum* CECT5716 for preventing GIs in infants from birth to one year of life. Additionally, this supplementation has been demonstrated to be well tolerated and without adverse effects. Nevertheless, given the study population, more evidence is necessary. Further studies including groups with a larger number of infants and longer time periods are recommended for applying specific recommendations. Follow-up studies will also be useful to guarantee the effect and safety. Only Gil-Campos et al. performed follow-up to evaluate long-term effects after three years [58]. The authors declared that early administration was safe, with no differences in infection incidence in comparison to control formula during the follow-up period. Thus, future RCTs should include subsequent monitoring after the intervention.

## 7. Conclusions

The findings of this systematic review and meta-analysis suggest a beneficial effect of *L. fermentum* CECT5716, a probiotic strain naturally found in breast milk, on the prevention of GIs in infants up to 12 months old. The use of a milk formula containing doses from 1 × 10^9^ to 8.4 × 10^8^ cfu/day of this probiotic reduces the incidence of diarrhea events without causing adverse events. As only three RCTs have been performed thus far, further studies including a larger number of infants and a longer time period should be performed to determine whether the use of this strain during an early stage of life is an efficient preventive measure to reduce the incidence of GIs and to address specific recommendations.

## Figures and Tables

**Table 1:** Summary of characteristics of included studies.

Authors (Year)	Study Design	Setting	N	Sample	Intervention	Time of Intervention	Findings Summary
Gil-Campos et al. (2012)	RCT, double blind, controlled, multi-center	Hospital Virgen de las Nieves (Granada, Spain), Hospital Reina Sofía (Córdoba, Spain), Hospital Carlos Haya (Málaga, Spain)	Randomized: 137 (IG:66, CG:71) Analyzed at the end of the study: 121 (IG:61, CG:60)	Healthy 1-month-old infants with exclusively formula-fed	Intervention: Standard powdered infant formula ^a^ with GOS supplemented or not with a probiotic strain. Follow up: 4 visits Experimental product and dose *L. fermentum* CECT5716 (8.4 × 10^8^ cfu/day) ^b^	From 1 to 6 months old	IG: ↓ incidence of GI (IR: 0.082 ± 0.037) vs. CG (IR: 0.283 ± 0.068)
Maldonado et al. (2012)	RCT, double blind, controlled, multi-center	Hospital San Cecilio (Granada, Spain), Hospital Virgen de las Nieves (Granada, Spain), Hospital de Poniente (El Ejido, Almeria, Spain)	Randomized: 215 (IG:117, CG:98) Analyzed at the end of the study: 188 (IG:97, CG:91)	Healthy 6-month-old infants, exclusively formula-fed	Intervention: Standard powdered infant formula ^a^ with GOS supplemented or not with a probiotic strain. Follow up: 3 visits Experimental product and dose: : *L. fermentum* CECT5716 (2 × 10^8^ cfu/day)	From 6 to 12 months old	IG: ↓ incidence of GI (IR: 0.196 ± 0.51) vs. CG (IR: 0.363 ± 0.53)
Maldonado et al. (2019)	RCT, double blind, controlled, multi-center	Hospital Virgen de las Nieves (Granada, Spain), Hospital Reina Sofía (Córdoba, Spain), Pediatric Clinic Roquetas de Mar (Almeria, Spain), Pediatric Clinic Cristo de la Salud (Granada, Spain), 7 Pediatric Services from the Andalusian Public Health System (Spain)	Randomized: 160 (IG1:83, CG:77) Analyzed per-protocol: 126 (IG1:65, CG:61)	Healthy 1-month-old infants, exclusively formula-fed	Intervention: Standard powdered infant formula ^a^ supplemented or not with a probiotic strain. Follow up: 6 visits Experimental product and dose: : *L. fermentum* CECT5716 (1 × 10^9^–8 × 10^8^ cfu/day) ^c^	From 1 to 12 months old	IG: ↓ incidence of GI (IR: 0.385 ± 0.077) vs. CG (IR: 0.689 ± 0.106)

Abbreviations: cfu, colony-forming units; RCT, randomized clinical trial; GOS, galactooligosaccharides; IG, intervention group; CG, control group; IR, incidence rate; GI, gastrointestinal infection. ^a^ The standard powdered infant formula had a nutritional composition in accordance with current EU regulations for both starter and follow-on formula. ^b^ Data kindly provided by the authors. ^c^ The daily consumption of the formula corresponded to an average dosage of probiotic bacteria of 1 × 10^9^ cfu/day up to 6 months and 7–8 × 10^8^ cfu/day between 6 and 12 months.

## Supplementary Materials

The following are available online at https://www.mdpi.com/article/10.3390/microorganisms9071412/s1, Supplementary Material S1: Preferred Reporting Items for Systematic Reviews and Meta-Analyses (PRISMA) statement, Supplementary Material S2: Detailed information of the search strategies.
